# Correction: Diffusion of methyl oleate in hierarchical micro-/mesoporous TS-1-based catalysts probed by PFG NMR spectroscopy

**DOI:** 10.1039/c8ra90098a

**Published:** 2018-11-30

**Authors:** Muslim Dvoyashkin, Nicole Wilde, Jürgen Haase, Roger Gläser

**Affiliations:** Institute of Chemical Technology, Universität Leipzig Linnéstr. 3 04103 Leipzig Germany muslim.dvoyashkin@uni-leipzig.de; Felix-Bloch-Institut, Universität Leipzig Linnéstr. 5 04103 Leipzig Germany

## Abstract

Correction for ‘Diffusion of methyl oleate in hierarchical micro-/mesoporous TS-1-based catalysts probed by PFG NMR spectroscopy’ by Muslim Dvoyashkin *et al.*, *RSC Adv.*, 2018, **8**, 38941–38944.

An error was present in the published article in the final sentence of the Abstract. The correct sentence should read as follows:

Additionally, the methodological capability of PFG NMR for measuring self-diffusion coefficients of long-chain hydrocarbons (up to C19) confined to narrow mesopores of catalytically active materials is demonstrated for the first time.

The Royal Society of Chemistry apologises for these errors and any consequent inconvenience to authors and readers.

## Supplementary Material

